# Correlation between the immuno-virological response and the nutritional profile of treatment-experienced HIV-infected patients in the East region of Cameroon

**DOI:** 10.1371/journal.pone.0229550

**Published:** 2021-05-13

**Authors:** Aissatou Abba, Joseph Fokam, Rachel Simo Kamgaing, Junie Flore Yimga, Aude Christelle Ka’e, Alex Durand Nka, Michel Carlos Tommo Tchouaket, Collins Ambe Chenwi, Ezechiel Ngoufack Jagni Semengue, Alexis Ndjolo, Samuel Martin Sosso

**Affiliations:** 1 Chantal BIYA International Reference Centre for Research on HIV/AIDS Prevention and Management (CIRCB), Yaoundé, Cameroon; 2 School of Health Sciences, Catholic University of Central Africa, Yaoundé, Cameroon; 3 Faculty of Health Sciences, University of Buea, Buea, Cameroon; 4 Faculty of Medicine and Biomedical Sciences (FMBS), University of Yaoundé I, Yaoundé, Cameroon; Lund University, SWEDEN

## Abstract

**Background:**

HIV management remains concerning and even more challenging in the frame of comorbidities like malnutrition that favors disease progression and mortality in resource-limited settings (RLS).

**Objective:**

To describe the nutritional parameters of antiretroviral therapy (ART) recipients (without nutritional support) with respect to CD4 count and virological failure.

**Methods:**

A cross-sectional study was conducted from October to December 2018 among 146 consenting participants enrolled in two health facilities of the East-Region of Cameroon. Socio-demographic data, basic clinical information and treatment history were collected; blood samples were collected by venipuncture for laboratory analysis (HIV-1 viral load, CD4 Tcells measurement and biochemical analysis) performed at the “Chantal Biya” International Reference Center”, Yaounde, Cameroon. The nutritional profile was assessed by using anthropometric and biochemical parameters. Data were analyzed using Excel 2016, Graph pad prism version 6; Spearman correlation and Kruskal-Wallis test were used; with p<0.05 considered statistically significant.

**Results:**

Median [IQR] age was 42 [33–51] years, 76.0% (111/146) were female and median [IQR] duration on ART was 54 [28–86] months. Of these participants, 11.6% (17/146) were underweight based on the body mass index and 4.7% (7/146) were at the stage of advanced weight loss. According to immunovirological responses, 44.5% (65/146) were immunocompromised (CD4<500 cell/μl) and 75.3% (110/146) had an undetectable viremia (<40 copies/mL). CD4 count inversely correlated with total protein concentration (r = -0.18, p = 0.005**). Viremia was inversely correlated with albumin (r = -0.21; p = 0.047*), nutritional risk index (r = -0.28; p = 0.013*), total cholesterol (r = -0.27; p = 0.007**), and positively correlated with total protein (r = 0.27; p<0.001**) concentrations.

**Conclusion:**

In this RLS, with patients having about five years of ART-experience, malnutrition appears to be driven mainly by a poor BMI, indicating that about one of ten patients falls within this severe condition. However, the largely normal nutritional profiles should be interpreted with caution, considering local realities and food support programs in place. The present outcomes highlight the need for monitoring nutritional status of people receiving ART in RLS, toward the design of optimal food interventions.

## Introduction

The human immunodeficiency virus (HIV) targets the immune system and weakens the surveillance by the body’s own defense system against cancer cells and infections, which in turns leads to susceptibility of HIV infected individuals to a wide range of infections normally cleared by the immune system of a healthy/immunocompetent individual [1]. HIV can therefore cause several health complications including opportunistic infections, oxidative stress, wasting syndrome, as well as malnutrition [2].

Malnutrition is one of the major complications of HIV infection [3] and has been recognized under the banner of ‘wasting syndrome’ as a significant prognostic factor of disease progression [4]. Of note, malnutrition (essentially focusing on under-nutrition) is a combination of factors that entails insufficient micronutrients, proteins and energy, exacerbated by frequent infections or disease conditions [5]. Specifically, under-nutrition impairs the immune system mechanism and thus weakens the host response against microorganisms. The consequence of this impairment is an increase in both incidence and severity of infections within the affected individual [6, 7]. Particularly, HIV infection and insufficient nutritional intake are parts of a vicious cycle that contributes to immunodeficiency and poor health outcomes [8]. Regarding the classification of global acute malnutrition (GAM) at population-level, the United Nations guidelines recommend that a threshold above 10% of GAM is considered as a high-level of severity, which in turns refers to a high public health concern that requires immediate actions [9]. Of relevance, evidence on the threshold of GAM, among vulnerable population such people living with HIV, remains largely unknown for informed decision-making.

HIV/AIDS and malnutrition seemingly have a synergistic interaction within the host, characterized by interaction between a poor nutritional status and HIV infection (driven by progressive degradation of immune system). This leads to the development of opportunistic infections and increases the risks of malignancies, CD4 T-cell depletion and death [10, 11]. In effect, malnutrition increases the risk of HIV pathogenesis, while HIV in turn triggers malnutrition by depleting the immune system from nutrient intake, absorption and utilization [12]. This complex triangulation mechanism, between malnutrition, the immune system and HIV infection, elicits immune system dysfunctions, increases vulnerability of the host to infection, and intensifies the severity of malnutrition [13]. Consequently, these observations suggest an unmet need among HIV-infected individuals, which may require informed food interventional programs for either corrective or preventive actions with respect to a specific micronutrient deficiency, especially in resource-limited settings (RLS) where malnutrition is common [14].

Progress in scaling-up HIV treatment (23.3 million on antiretroviral therapy [ART], representing 62% of the global coverage) have reduced associated mortality and morbidity, thus making HIV a chronic infection, even in sub-Saharan African (SSA) countries where 70% of the global epidemics is concentrated [15–18]. Thus, for an enhanced performance of ART programs, it is postulated that a desirable nutritional profile would be a surrogate of an improved treatment outcome among people living with HIV (PLHIV) [19]. Such strategy suggests that improved attention to diet and nutrition could normalize protein profiling, fatty acids, copper and iron, which in turn would harness the immune function and subsequently optimize ART acceptability, adherence and effectiveness [20, 21].

In spite of the declining burden of HIV at country-level (5.4% in 2004 to 2.7% in 2019), Cameroon is still experiencing a generalized epidemiology. The national strategy for the fight against HIV/AIDS in Cameroon recommends a nutritional supplement to subside the current 14.1% persistent rate of malnutrition faced by PLHIV, which intends to support ART response [22, 23]. Interestingly, the East region of Cameroon has a very high burden of HIV infection (5.6%), 43.01% patients are on ART [24, 25], while up to 13.7% of these patients are known to be undernourished [23]. Thus, these high rates of both HIV infection and malnutrition call for further investigations to improve the management of this comorbidity in similar settings within SSA.

We therefore sought to describe the nutritional parameters of ART recipients in real-life (without nutritional support) with respect to CD4 count and virological failure in the East-region of Cameroon.

## Materials and methods

### Study design, enrollment procedure and eligibility criteria

A prospective, cross-sectional and analytical study was carried-out among ART-experienced HIV-infected patients at two heath facilities in the East region of Cameroon, Bertoua Regional Hospital (BRH); and Nkolbikon Catholic Health Center (NCHC), during three months (from October through December 2018).

A standard questionnaire (S1 File) was administered to PLHIV, by interviewers were trained on the study protocol, covering socio-demographic data, treatment history and food habits (food habits were determined based on information given by the participant about their daily food intake during the past three days), as well as basic clinical information and all biological parameters performed. Interviewers were trained to collect all these information from participants.

Following a convenience sampling strategy, participants were enrolled consecutively based on the following inclusion criteria: (1) having a confirmed HIV-positive result; (2) be on ART for at least six months; (3) be registered in one of the study sites; and (4) aged 15 years and above. Those excluded from our study were: (1) patient enrolled in a nutritional program or food-interventional project, or (2) those having incomplete data related to any of the study parameters. It is worth noting that excluding patients already in a nutritional program helps in limiting selection bias and in ensuring generalizability of the findings to the target population.

### Phlebotomy and sample shipment

Blood sample was collected (in dry and ethylene diamine tetra-acetic acid test tubes of 4 ml) by venipuncture with the help of trained phlebotomists. After collection, blood samples were transferred from the sampling sites to the BRH laboratory for packaging and shipment. Only dry test tubes were centrifuged and separated in aliquots for biochemical analysis. After centrifugation, racks of all samples were placed in ice packed isothermal bags, and samples were then sent for laboratory analysis to the study reference institution (Chantal BIYA International Reference Center for Research on HIV/AIDS prevention and management), located in Yaoundé, the capital city of Cameroon.

### Clinical and laboratory procedures

At the study site, anthropometric parameters were collected from each participant immediately after the interview using a scale (use to collect weight), a measuring board (used to collect height) and the patient’s report (used for the patient’s treatment history and basic clinical information including usual weight).

CD4 and CD8 cell count were performed using the Cyflow Counter-Sysmex Partec as per the manufacturer’s instructions with results reported as the number of positive cells per microliter of blood (http://www.nsplucknow.com/pdf/CyFlow_Counter_Bro_EN.pdf). CD4 results were then interpreted as follows: no immunodeficiency (above or equal to 500); mild immunodeficiency (between 350 and 499); advanced immunodeficiency (between 200 and 349) and severe immunodeficiency (below 200) [26]. Viral load measurement was performed using the Abbott m2000RT Real Time PCR as-per the manufacturer’s instructions (Abbott Laboratories, USA), with a lower detection threshold of 40 HIV-1 RNA copies/mL and an upper detection threshold of 10,000,000 copies/mL (www.abbottmolecular.com/products/infectious-diseases/realtime-pcr/hiv-1-assay).

The nutritional profile of participants was evaluated based on biochemical parameters: albumin, calcium, glucose, iron, magnesium, total cholesterol, triglycerides, total protein and anthropometric parameters of which the body mass index (BMI), nutritional risk index (NRI) and weight loss percentage (WLP). Biochemical analysis was performed using BT-3000 Plus as per manufacturer’s instructions (https://www.chema.com/chema/automation_it_files/Biotecnica%20BT3000.pdf).

BMI was defined as an indicator of chronic energy malnutrition and was calculated by dividing the weight (in Kg) by the height squared (in square meter):

$$BMI=[\frac{Weight}{{Height}^{2}}];$$

[27]

The NRI, a nutritional status assessment index recommended in national nutritional programs, was estimated by the following formula:

$$NRI=(1.519\times serum\;albumin,\frac{g}{dL})+\frac{41.7\times present\;weight(kg)}{ideal\;body\;weight(kg)};$$

[28]

WLP is used to assess the risk of malnutrition and determined by the formula

$$WLP=(\frac{Weight\;Lost}{Usual\;Weight})\times 100$$

WeightLost=UsualWeight-Presentweight;

[29]

According to each of the studied nutritional parameters, the level of malnutrition was defined as severe if the global acute malnutrition (GAM) rate was above 10%, as per the United Nations guidelines [9].

### Statistical analysis

Collected data were entered on Microsoft Excel 2016 sheets and used for basic analysis (median, IQR) The software GraphPad Prism version 6.0. was used for correlation tests. Correlation analysis were performed using Spearman correlation and Kruskal-Wallis test was used to analyze the median (IQR) of nutritional parameters according to categories of CD4 (<200, 200–349, 350–499, >500 cells/μL) and VL (<40, 40–1000, >1000 copies/mL). A statistical significance was considered with p<0.05. Dependent variables were immune status and viremia, while independent variables were anthropometric and biochemical parameters.

### Ethical considerations

The study was conducted in compliance with the core principles of the Helsinki declaration: an administrative authorization was issued; ethical clearance was obtained from the National Ethics Committee for Research on Human Health (ref N^0^ 2018/06/1055/CE/CNERSH/SP); written informed consent were obtained from all the participants; data were processed using unique identifiers to ensure confidentiality; laboratory results were returned to participants for possible benefits in their clinical management; and counseling on good nutritional habits and healthy lifestyles was provided to all (good nutritional habits were provided by a well-experienced clinician in the field of nutrition management. Generated data from each patient were tested for normality).

## Results

### Socio-demographic and clinical characteristics of the population

Of the total of 146 participants enrolled with complete data, 88.4% (n = 129) at Bertoua regional hospital (BRH) and 11.6% (n = 17) at Nkolbikon Catholic Health Center (NCHC). Among them, 76.0% (n = 111) were females giving a sex ratio (female/male) of 3. The median age was 42 [IQR: 33–51] years (Table 1).

**Table 1:** Socio-demographic data and nutritional parameters.

Variables	Effective			Percentage (%)		
**Health Center**						
BRH	129			88.4		
NCHC	17			11.6		
**Gender**						
Male	35			23.9		
Female	111			76.0		
**Economic status per day (Francs CFA)**						
<500	8			5.5		
500–1000	27			18.5		
1000–5000	107			73.3		
>5000	4			3.7		
**Average of meals per day**						
1 meal	12			8.2		
2 meals	72			49.3		
3 meals	54			36.9		
4 meals	8			5.5		
**BMI**						
>25.0	42			28.7		
18.5–25.0	87			59.6		
<18.5	17			11.6		
**NRI**						
>100	133			91.1		
97.5–100	5			3.4		
83.5–97.5	8			5.5		
<83.5	0			0.0		
**WLP**						
0%	89			60.9		
-5%-0%	50			34.2		
“-10%”–“-5%”	7			5.8		
< -10%	0			0.0		
**Biochemical parameters**	High	Normal	Low	High	Normal	Low
Albumin: 37–53 g/l	6	136	4	4.1	93.2	2.7
Calcium: 86–103 mg/l	13	105	28	8.9	71.9	19.2
Glucose: 0.7–1.15 g/l	4	111	31	2.7	76.0	21.2
Iron: 0.5–1.6 mg/l	37	90	19	25.3	61.6	13.0
Magnesium: 13–21 mg/l	120	24	2	82.2	16.4	1.4
Total cholesterol: 1.4–2.0 g/l	5	111	30	3.4	76.0	20.5
Total protein: 63–83 g/l	29	115	2	19.9	78.7	1.4
Triglycerides: 0.0–2.0 g/l	9	137	0	6.2	93.8	0.0

BRH: Bertoua regional hospital; NCHC: Nkolbikon Catholic Health Center; BMI: Body Mass Index; NRI: Nutritional Risk Index; WLP: Weight Loss Percentage.

The median duration on ART was 54 [IQR: 28–86] months. All participants were on first-line ART regimen consisting of two nucleoside reverse transcriptase inhibitors (NRTIs) and one non-NRTI (NNRTI).

### Nutritional profile

Of the 146 participants, BMI results revealed that only a proportion of 11.6% (n = 17) was undernourished. According to NRI, 91.1% (n = 133) were normal, while referring to WLP, 60.9% (n = 89) had a normal weight. Only 2.7% (4) of participants were hypoalbuminemic, 19.9% (n = 29) hyperproteinemic and 20.5% (n = 30) hypocholesterolemic.

The median [IQR] CD4+ T cell lymphocyte count was 547 [385–759] cells/μl, and 44.5% (n = 65) were immunocompromised (<500 cells/μl). The median [IQR] of low CD4 (below 200 cell/μl) according to BMI was 21.2 [17,9–24.5] and to WLP was 1.7 [-1.31–4.9]%. The majority (75.3%) of the 146 study participants had a controlled of viral replication (<40 copies/mL) while 12.3% (n = 18) were experiencing virological failure (>1000 copies/mL). The median [IQR] of high viremia (≥1000 copies/mL) according to total cholesterol was 1.5 [1.3–1.6] g/l and to WLP was 1.6 [-1.4–6.2]% (Table 2).

**Table 2:** Variation of immuno-virological data according to the median distribution of nutritional parameters.

	CD4 (cell/*μ*L)					Viremia (copies/mL)			
	<200	[200–349]	[350–499]	>500	KWT	<40	[40–1000]	≥1000	KWT
	n = 4	n = 25	n = 36	n = 81		n = 111	n = 17	n = 18	
	Median	Median	Median	Median	P	Median	Median	Median	P
	[IQR]	[IQR]	[IQR]	[IQR]		[IQR]	[IQR]	[IQR]	
Albumin: g/l	39	46	45	45	0.234	45	46	43	0.013*
(37–53)	[35–44]	[42–49]	[43–48]	[43–47]		[43–48]	[45–48]	[39–45]	
BMI	21.2	23.2	22.5	22.5	0.676	23.1	21.6	22.8	0.288
(18.5–25.0)	[17.9–24.5]	[21.1–27.1]	[21.2–25.0]	[20.8–26.7]		[21.0–26.4]	[20.3–22.6]	[20.2–24.6]	
Calcium: mg/l	94	91	92	93	0.769	92	95	92	0.191
(86–103)	[87–95]	[87–95]	[86–97]	[88–97]		[88–97]	[91–97]	[84–94]	
Glucose: g/l	0.8	0.8	0.8	0.8	0.916	0.8	0.8	0.8	0.92
(0.7–1.15)	[0.8–1.0]	[0.7–0.8]	[0.7–0.9]	[0.7–0.9]		[0.7–0.9]	[0.7–0.8]	[0.7–0.8]	
Iron: mg/l	1.0	0.8	1.1	1.1	0.115	1.1	0.9	0.9	0.432
(0.5–1.6)	[0.7–1.2]	[0.5–1.1]	[0.8–2.2]	[0.7–1.7]		[0.6–1.8]	[0.7–1.1]	[0.5–1.6]	
Magnesium:	24	24	25	24	0.969	24	25	24	0.377
mg/l (13–21)	[23–27]	[22–27]	[22–27]	[22–27]		[22–27]	[24–27]	[21–26]	
NRI	103.3	114.0	112.4	112.9	0.122	114.2	111.4	106.5	0.047*
(83.5–100)	[96.1–105.6]	[103.4–119.5]	[105.5–118.1]	[106.0–118.9]		[106.1–119.1]	[104.2–114.8]	[101.7–111.2]	
Total Cholesterol: g/l (1.4–2.0)	1.6	1.8	1.8	1.7	0.635	1.8	1.7	1.5	0.007**
	[1.2–1.8]	[1.5–2.1]	[1.4–2.2]	[1.4–2.0]		[1.4–2.2]	[1.3–1.9]	[1.3–1.6]	
Total protein: g/l (63–83)	98	78	78	76	0.005**	76	79	85	<0.001**
	[92–107]	[75–85]	[75–80]	[71–81]		[73–81]	[76–82]	[79–97]	
Triglycerides: g/l (0.0–2.0)	1.2	0.9	0.9	0.9	0.722	0.8	1.1	1.0	0.353
	[0.5–1.5]	[0.3–1.1]	[0.5–1.2]	[0.6–1.2]		[0.5–1.8]	[0.9–1.3]	[0.9–1.2]	
WLP	1.7%	0.0%	2.2%	1.9%	0.827	1.8%	1.1%	1.6%	0.707
(>0%)	[-1.3–4.9]%	[-1.4–8.6]%	[-1.7–9.7]%	[0.0–7.9]%		[-1.2–8.6]%	[0.0–7.7]%	[-1.4–6.2]	

BMI: Body Mass Index; NRI: Nutritional Risk Index; WLP: Weight Loss Percentage; N: Effective; KWT: Kruskal-Wallis test; P: P Values.

### Correlation between immuno-virological parameters and nutritional profile

**Fig 1:**
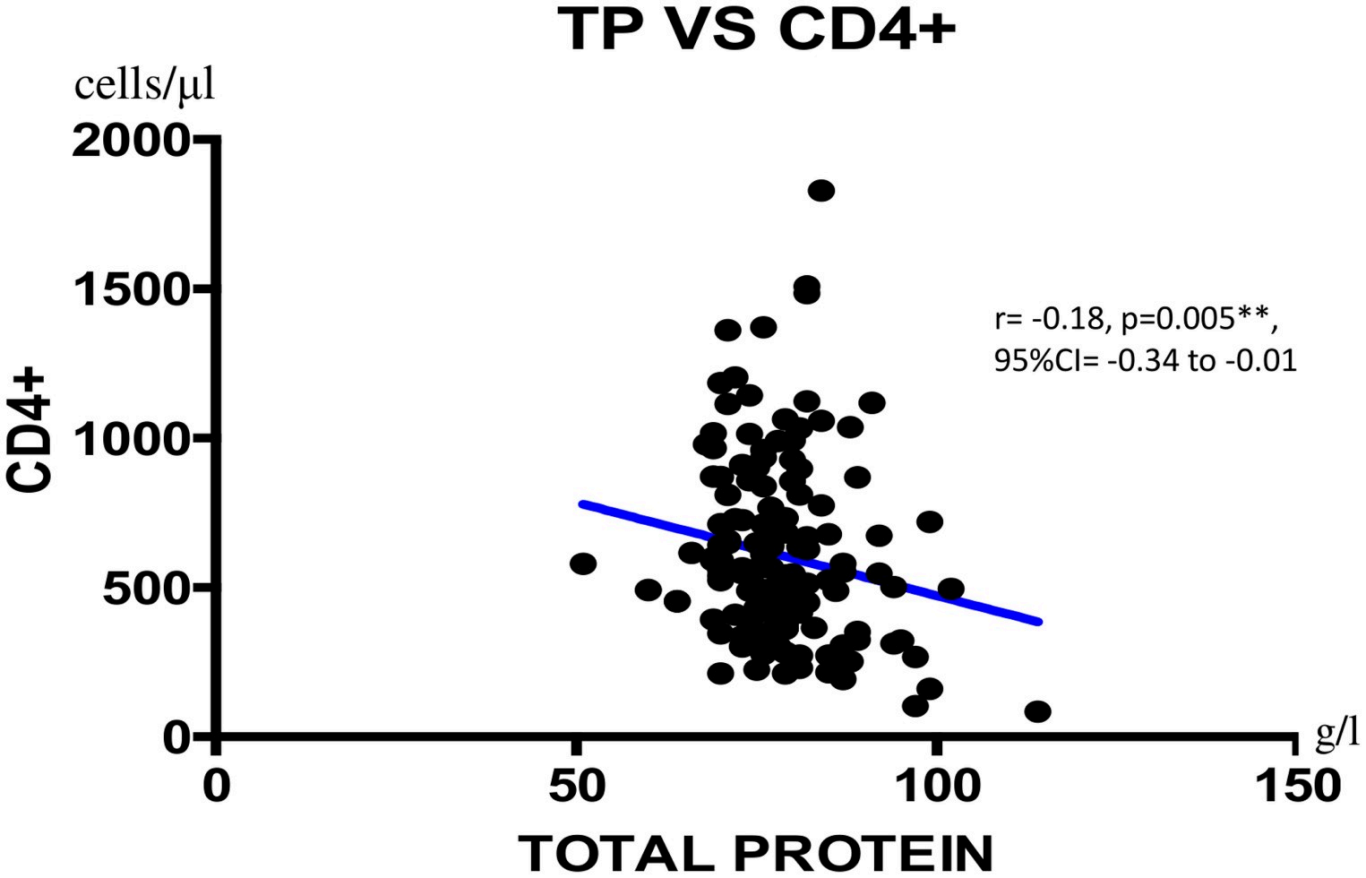
Correlation between LTCD4+ and total protein. TP = Total protein; CI: Confidence Interval.

**Fig 2:**
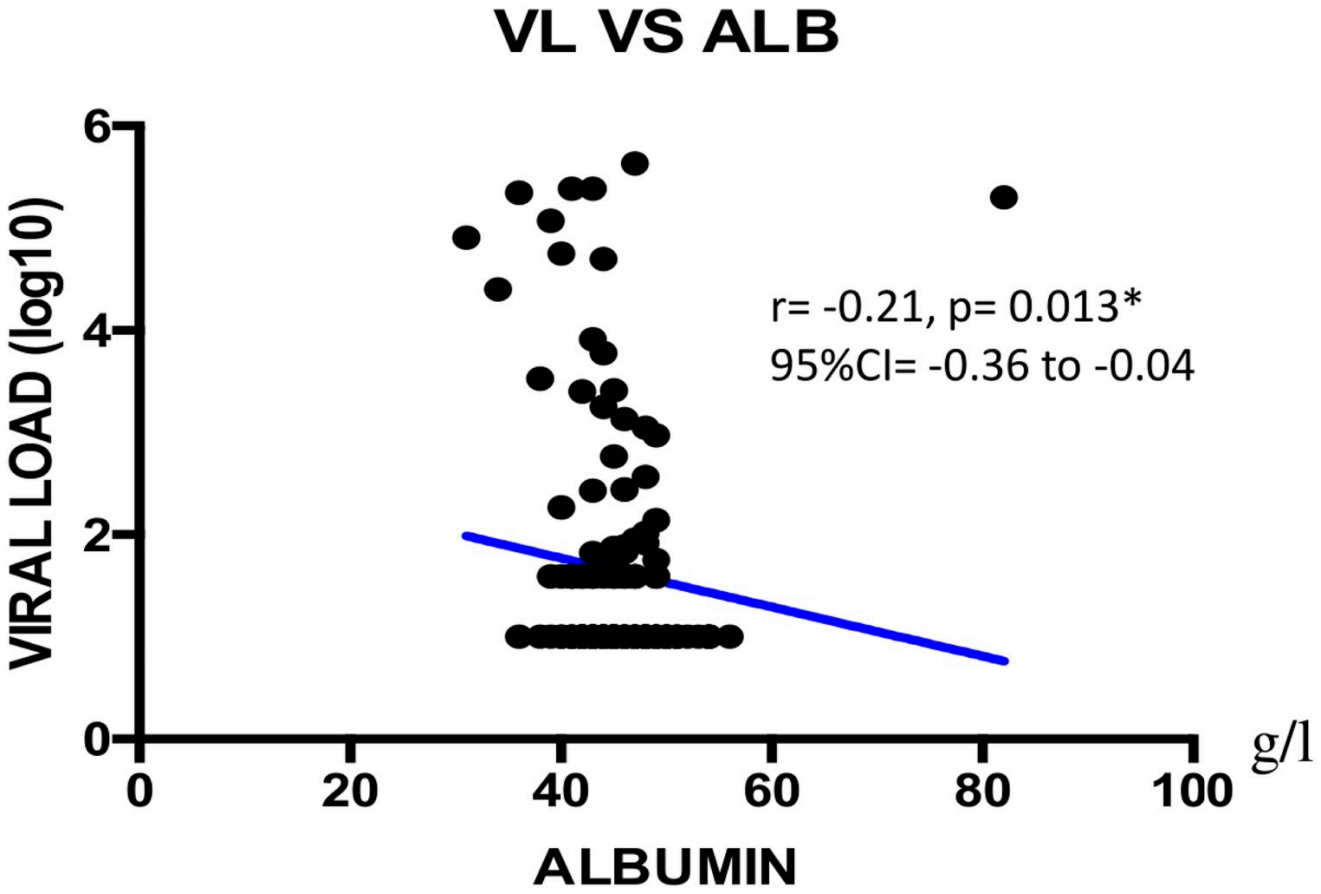
Correlation between viral load and albumin. VL = viral load; CI: Confidence Interval.

**Fig 3:**
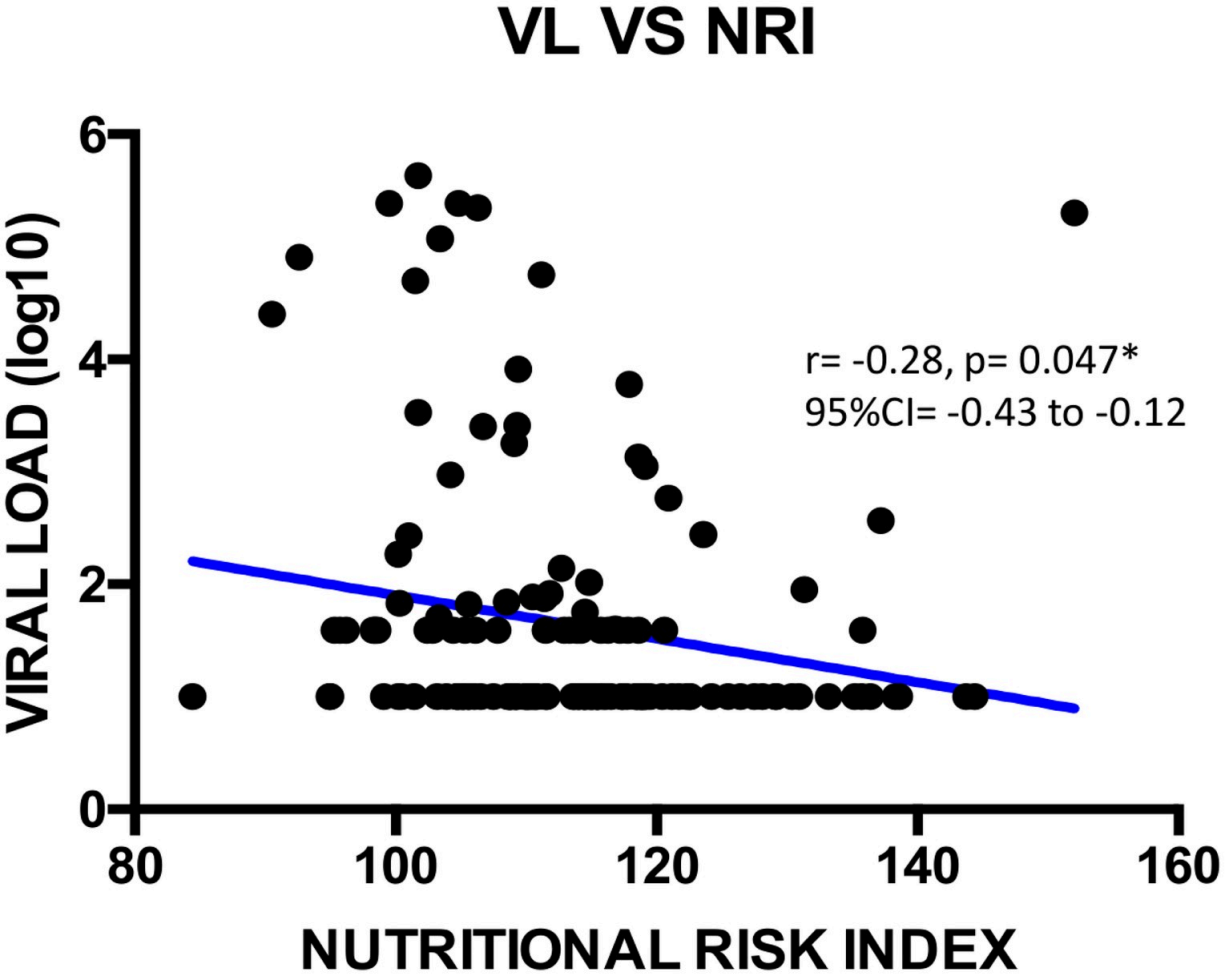
Correlation between viral load and NRI. VL = viral load; NRI = nutritional risk index; CI: Confidence Interval.

**Fig 4:**
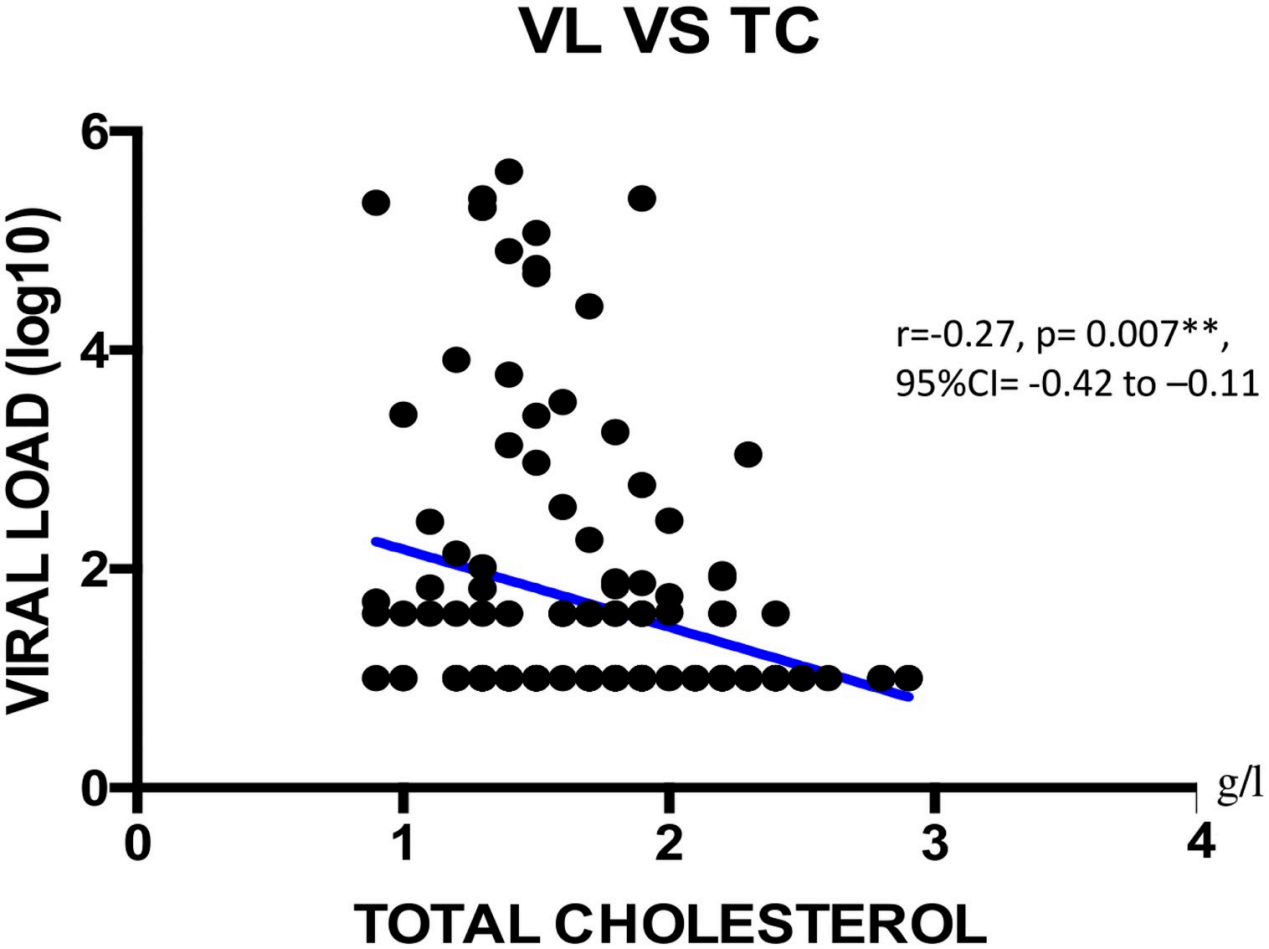
Correlation between viral load and total cholesterol. VL = viral load; TC = total cholesterol; CI: Confidence Interval.

**Fig 5:**
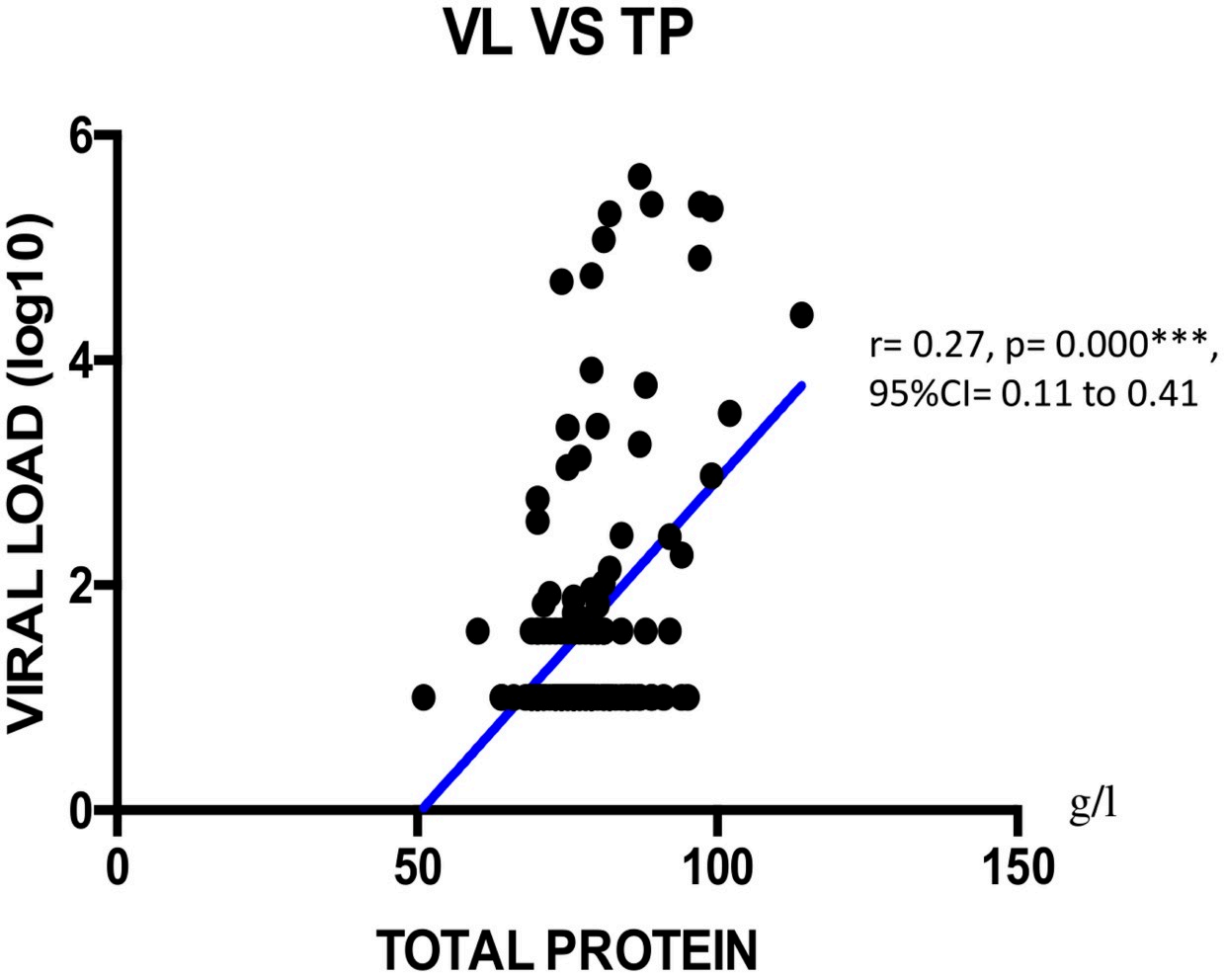
Correlation between viral load and total protein. VL = viral load; TP = Total protein; CI: Confidence Interval.

We found a negatively weak correlation between CD4+ T cells count and total protein concentration (r = -0.18; **p = 0.005; 95%CI = -0.34 to -0.01) as shown on Fig 1. Viral load showed a negatively weak correlation with albumin (r = -0.21; *p = 0.013; 95%CI = -0.36 to -0.04), with NRI (r = -0.28; *p = 0.047; 95%CI = -0.43 to -0.12), with total cholesterol (r = -0.28, **p = 0.007; 95%CI = -0.42 to -0.11) as shown Figs 2–4 respectively, and a positively weak correlation with total protein (r = 0.28, **p<0.001); 95%CI = 0.11 to 0.41) shown Fig 5.

## Discussion

The present study intended to correlate ART response and the nutritional profile among patients receiving ART within the Cameroonian context. Our findings revealed that the majority of our study participants were predominantly female, as reported by previous studies in the country. Similarly, the median age from our study also falls within the age range of the predominant population of PLHIV in Cameroon [23, 30, 31], thereby underscoring representativeness of the Cameroonian target population.

Regarding the analysis of anthropometric measurements, about 1 out of 10 participants were undernourished as defined by a poor BMI, suggesting that this poor nutritional status in the Cameroonian context would represent a high public health concern among patients receiving ART. Interestingly, similar findings were recently reported among ART-compliant recipients in Ethiopia; this underscores the fact that beyond adherence to an effective ART-regimen, malnutrition remains a threat that deserves special considerations in the SSA context [32]. Furthermore, even though weight loss was found in 40% of our participants, this does not represent a substantial decline of body weight. In fact, 34% out of the 40% reported had experienced weight loss <5% of body weight [33, 34]. Poverty was likely a contributing factor to weight loss, this as indicated by the poor daily ration observed in our study (i.e. about three quarters living on a daily ration ranging between $2 and $10 per day, and only half could afford 2 meals per day). Consequently, the reported WLP also highlights the financial constraint HIV could have on food insecurity among Cameroonian families [35].

Regarding the analysis of micronutrients, most nutritional parameters were within normal ranges. Of note, the normal total protein profile of the large majority of our population indicates up-regulation of antibody production in an attempt to compensate ongoing immunodeficiency [36]. This is particularly plausible as recent findings suggest that diet can influence systemic markers of immune function and inflammation [37]. On the same line, about half of our participants had a normal immunity, similar to previous reports in Cameroon [38, 39]. Of relevance, the negatively weak correlation between CD4+ T-cells and total protein was previously reported by Lyer et al. in a population of black Americans [40, 41]. Overall, this favorable immune status (CD4+) further reflects the good ART response commonly found among patients receiving ART in Cameroon (~80% viral suppression) [30, 42].

Regarding ART response, the majority of participants achieved viral undetectability and viral suppression similar to previous reports [38, 43]. Regarding the correlation between ART response and the studied nutritional parameters, albumin, NRI, total protein and total cholesterol were independent factors correlating with plasma viral load. Of note, albuminemia is likely to vary with inflammatory response, renal and hepatic functions in the course of HIV infection [44]. Furthermore, albumin and pre-albumin are often used as indicators of nutritional status, while albumin represents a key component of the NRI that serves for an in-depth evaluation of the nutritional profile [45]. On the one hand, the negative correlation between the NRI and plasma viral load suggests that correcting for under-nutrition would improve on anthropometric measurements, thus contributing in the control of viral replication and an improved health condition of PLHIV [28]. On the other hand, total protein is mainly an inflammatory marker and is not a sign of malnutrition. It is known to be associated with HIV disease severity, viremia and low CD4 count. This is clearly shown in Table 2 with normal levels for all groups except CD4 <200 which is higher in total protein [43]. Furthermore, it seems at least as likely that socioeconomically vulnerable individuals with food insecurity also have worse ART adherence [46–48], while an increased viral load is also known to impair cholesterol level [38]. This observation suggests an interaction between HIV and cholesterol, which might lead to metabolic abnormalities (lipodystrophy, dyslipidemia, diabetes mellitus, and insulin resistance) prone by HIV itself and/or antiretroviral agents [44]. Finally, correlation between viral load and total cholesterol suggests that the latter may play a role in HIV life cycle owing to lipid functions in viral entry, uncoating, replication, protein synthesis, assembly, budding and infectivity [49, 50].

The present study had some limitations. Of note, the geographical coverage was limited to a single region of Cameroon and the enrollment was non-randomized, which limit somehow the generalizability of our findings. Also, it would have been insightful to assess pro and anti-inflammatory markers with regards to ART response and variability of the nutritional status. It is plausible that the largely normal nutritional profiles found in our study could be due to the fact that patients experiencing malnutrition locally would have been enrolled into the nutrition interventional program onsite, and therefore excluded from our study population (suggesting an underestimation of the severity in real-life). Furthermore, only self-reported adherence has been used to evaluate effects of ART on participants; the cross-sectional design of our study does not give room for causality assessment of HIV on malnutrition, which underscores the relevance of conducting cohort-studies for a deeper understanding of the potential interaction between the two. Moreover, our findings should be cautiously interpreted because of the large number (n = 22) of statistical tests performed (2 exposures [CD4 and VL] across 11 nutritional outcomes).

## Conclusion

In this RLS, with patients having about five years of ART-experience, malnutrition appears to be driven mainly by a poor BMI, indicating that about one of ten patients falls within this severe condition. However, the largely normal nutritional profiles should be interpreted with caution, considering local realities and food support programs in place. The present outcomes highlight the need for monitoring nutritional status of people receiving ART in RLS, toward the design of optimal food interventions.

## Supporting information

S1 Table Socio-demographic data and nutritional parameters. BRH: Bertoua regional hospital; NCHC: Nkolbikon Catholic Health Center, BMI: Body Mass Index; NRI: Nutritional Risk Index; WLP: Weight Loss Percentage.
(PDF)

S2 Table Variation of immuno-virological data according to the median distribution of nutritional parameters. BMI: Body Mass Index; NRI: Nutritional Risk Index; WLP: Weight Loss Percentage; N: Effective; KWT: Kruskal-Wallis test; P: P Values.
(PDF)

S1 File The questionnaire. (PDF)
