# Supplementary material for: Correlation between the immuno-virological response and the nutritional profile of treatment-experienced HIV-infected patients in the East region of Cameroon
Source: PLoS One. 2021 May 13;16(5):e0229550. doi: 10.1371/journal.pone.0229550 (PMC8118549; doi:10.1371/journal.pone.0229550)
Supplement: S1 Table — BRH: Bertoua regional hospital; NCHC: Nkolbikon Catholic Health Center, BMI: Body Mass Index; NRI: Nutritional Risk Index; WLP: Weight Loss Percentage. (PDF) [file pone.0229550.s001.pdf]

S1 **Table. Socio-demographic data and nutritional parameters.**

Variables	Effective	Percentage (%)
**Health Center**		
BRH	129	88.4
NCHC	17	11.6
**Gender**		
Male	35	23.9
Female	111	76.0
**Economic status per day (Francs CFA)**		
<500	8	5.5
500-1000	27	18.5
1000-5000	107	73.3
>5000	4	3.7
**Average of meals per day**		
1 meal	12	8.2
2 meals	72	49.3
3 meals	54	36.9
4 meals	8	5.5
**BMI**		
>25.0	42	28.7
18.5-25.0	87	59.6
<18.5	17	11.6
**NRI**		
>100	133	91.1
97.5-100	5	3.4
83.5-97.5	8	5.5
<83.5	0	0.0
**WLP**		
0%	89	60.9
-5%-0%	50	34.2
"-10%" – "-5%"	7	5.8
< -10%	0	0.0

Biochemical parameters	High	Normal	Low	High	Normal	Low
Albumin: 37-53 g/l	6	136	4	4.1	93.2	2.7
Calcium: 86-103 mg/l	13	105	28	8.9	71.9	19.2
Glucose: 0.7-1.15 g/l	4	111	31	2.7	76.0	21.2
Iron: 0.5-1.6 mg/l	37	90	19	25.3	61.6	13.0
Magnesium: 13 – 21 mg/l	120	24	2	82.2	16.4	1.4
Total cholesterol: 1.4-2.0 g/l	5	111	30	3.4	76.0	20.5
Total protein: 63-83 g/l	29	115	2	19.9	78.7	1.4
Triglycerides: 0.0-2.0 g/l	9	137	0	6.2	93.8	0.0

Legend: BRH: Bertoua regional hospital; NCHC: Nkolbikon Catholic Health Center;
BMI: Body Mass Index; NRI: Nutritional Risk Index; WLP: Weight Loss Percentage.
