# Supplementary material for: Correlation between the immuno-virological response and the nutritional profile of treatment-experienced HIV-infected patients in the East region of Cameroon
Source: PLoS One. 2021 May 13;16(5):e0229550. doi: 10.1371/journal.pone.0229550 (PMC8118549; doi:10.1371/journal.pone.0229550)
Supplement: S2 Table — BMI: Body Mass Index; NRI: Nutritional Risk Index; WLP: Weight Loss Percentage; N: Effective; KWT: Kruskal-Wallis test; P: P Values. (PDF) [file pone.0229550.s002.pdf]

S2 Table. Variation of immuno-virological data according to the median distribution of nutritional parameters.

	CD4 (cell/µL)					Viremia (copies/mL)			
	**<200**	**[200-349]**	**[350-499]**	**>500**	**KWT**	**<40**	**[40-1000]**	**≥1000**	**KWT**
	**n=4**	**n=25**	**n=36**	**n=81**		**n=111**	**n=17**	**n=18**	
	Median [IQR]	Median [IQR]	Median [IQR]	Median [IQR]	P	Median [IQR]	Median [IQR]	Median [IQR]	P
Albumin: g/l (37-53)	39 [35-44]	46 [42-49]	45 [43-48]	45 [43-47]	0.234	45 [43-48]	46 [45-48]	43 [39-45]	0.013*
BMI (18.5-25.0)	21.2 [17.9-24.5]	23.2 [21.1-27.1]	22.5 [21.2-25.0]	22.5 [20.8-26.7]	0.676	23.1 [21.0-26.4]	21.6 [20.3-22.6]	22.8 [20.2-24.6]	0.288
Calcium: mg/l (86-103)	94 [87-95]	91 [87-95]	92 [86-97]	93 [88-97]	0.769	92 [88-97]	95 [91-97]	92 [84-94]	0.191
Glucose: g/l (0.7-1.15)	0.8 [0.8-1.0]	0.8 [0.7-0.8]	0.8 [0.7-0.9]	0.8 [0.7-0.9]	0.916	0.8 [0.7-0.9]	0.8 [0.7-0.8]	0.8 [0.7-0.8]	0.92
Iron: mg/l (0.5-1.6)	1.0 [0.7-1.2]	0.8 [0.5-1.1]	1.1 [0.8-2.2]	1.1 [0.7-1.7]	0.115	1.1 [0.6-1.8]	0.9 [0.7-1.1]	0.9 [0.5-1.6]	0.432
Magnesium: mg/l (13-21)	24 [23-27]	24 [22-27]	25 [22-27]	24 [22-27]	0.969	24 [22-27]	25 [24-27]	24 [21-26]	0.377
NRI (83.5-100)	103.3 [96.1-105.6]	114.0 [103.4-119.5]	112.4 [105.5-118.1]	112.9 [106.0-118.9]	0.122	114.2 [106.1-119.1]	111.4 [104.2-114.8]	106.5 [101.7-111.2]	0.047*
Total Cholesterol: g/l (1.4-2.0)	1.6 [1.2-1.8]	1.8 [1.5-2.1]	1.8 [1.4-2.2]	1.7 [1.4-2.0]	0.635	1.8 [1.4-2.2]	1.7 [1.3-1.9]	1.5 [1.3-1.6]	0.007**

Total protein: g/l (63-83)	98 [92-107]	78 [75-85]	78 [75-80]	76 [71-81]	0.005**	76 [73-81]	79 [76-82]	85 [79-97]	<0.001**
Triglycerides: g/l (0.0-2.0)	1.2 [0.5-1.5]	0.9 [0.3-1.1]	0.9 [0.5-1.2]	0.9 [0.6-1.2]	0.722	0.8 [0.5-1.8]	1.1 [0.9-1.3]	1.0 [0.9-1.2]	0.353
WLP (>0%)	1.7% [-1.3-4.9]%	0.0% [-1.4-8.6]%	2.2% [-1.7-9.7]%	1.9% [0.0-7.9]%	0.827	1.8% [-1.2-8.6]%	1.1% [0.0-7.7]%	1.6% [-1.4-6.2]	0.707

Legend: BMI: Body Mass Index; NRI: Nutritional Risk Index; WLP: Weight Loss Percentage; N: Effective; KWT: Kruskal-Wallis test;
