# Supplementary material for: Correlation between the immuno-virological response and the nutritional profile of treatment-experienced HIV-infected patients in the East region of Cameroon
Source: PLoS One. 2021 May 13;16(5):e0229550. doi: 10.1371/journal.pone.0229550 (PMC8118549; doi:10.1371/journal.pone.0229550)
Supplement: S1 File — (PDF) [file pone.0229550.s003.pdf]

QUESTIONNAIRE

STUDY TITLE: Correlation between the immuno-virological response and the nutritional profile among treatment-experienced HIV-infected patients in the East-region of Cameroon

ID: ……………………………. FOSA: ………………………….…….
Surname: ……………………………..... Given names: ………………………………….
Date of birth (dd/mm/yyyy): ………………… Age (In years): …….......
Gender: ☐ M ☐ F ; if F **Pregnant?** : ☐ Yes ☐ No
Profession: ……………………………..…. Tel: …………………………...
Region: …………….. Division: ……………….... Birth town: ………………….
Quarter: …………………... Number of children: ……………..
Number of children with HIV: ………….. Health Facility :…………………………………

Current weight: …………….. Usual weight………….. Height (m) : ………………..

1. **Educational status**: ☐ No formal education ☐ Primary
   ☐ Secondary 1st cycle ☐ Secondary 2nd cycle ☐ University

2. **Marital status**: ☐ Single ☐ Cohabitation ☐ Married
   ☐ Divorced/separated ☐ Widow/Widower
3. **Ethnic origin**: …………………………………….

4. **Drinkable water source** : ☐ Protected : (tap, Drilling, mineral)
   ☐ Not protected (wells, streams, rain water)

5. **Alcohol consumption:** ☐ Occasional ☐ Regular ☐ Never

6. **Smoker?** ☐ Yes ☐ No
   If yes, number of cigarettes per day : ☐ <5 ☐ 5-10 ☐ 10-20 ☐ >20

7. **What can you say about your economic status per day** : ☐ < 500frs ☐ 500 - 1000
   ☐ 1000 - 5000 ☐ > 5000 frs

8. **Have you ever had a nutritional counselling** (During the management of your HIV status)? ☐ Yes ☐ No ☐ For another reason: …………
   If yes, how many counselling sessions have you had?
   - < 3 months? …………………………………………..
   - 3 - 6 months? ....
   - > 6 months? ……………………………………………

9. **Number of meals per day (average), snacks included.**
   ☐ 1 ☐ 2 ☐ 3 ☐ 4 ☐5 ☐ 6 ☐>7

10. **Have you ever been in a period of malnutrition**? ☐ Yes ☐ No ☐ I don't know
    If yes, when? …………………………………………………
    Which type of malnutrition? Underweight ☐ Overweight ☐ Obese ☐

11. Clinical examination:

☐ Fever ☐ Nausea ☐ Anorexia (loss of appetite)
☐ Diarrhea (☐ Yes ☐ No If yes, number of times per day ………)
☐ Vomiting (☐ Yes ☐ No If yes, number of times per day ………)

12. **Edema**: ☐ Absent ☐ Present
If present, localization: ☐ Legs ☐ Tibia ☐ Hands ☐ Face ☐ General

13. **Other symptoms and clinical signs**: ……………………………………………………………

14. **Other pathologies**: ☐ Yes ☐ No
If yes: ☐ Tuberculosis ☐ HBV ☐ HCV ☐ Malaria ☐ Typhoid Fever
☐ Diabetes Other…………………………………………………………..

15. **WHO clinical stage of the HIV infection**: ☐ 1 ☐ 2 ☐ 3 ☐ 4

16. **Have you lost weight during the past 3 months**? ☐ Yes ☐ No ☐ I don't know

17. **Have you ever taken** :

- Vit A : ☐ Yes ☐ No
- Other micronutrients: ☐ Yes ☐ No

18. **ARV treatment**:
Date of 1st HIV diagnosis: …… /…… /……
Mode of Infection: ☐ Vertical ☐ Horizontal

- HIV Treatment 1: ☐ …………….…………….… from …../…../….. to ..…/…../…..
- HIV Treatment 2: ☐……………………………… from …../…../….. to ..…/…../…..
- HIV Treatment 3: ☐...…………………………… from …../…../….. to ..…/…../…..

NOW, I WOULD LIKE TO ASK QUESTIONS ABOUT THE NOURISHMENT YOU MAY HAVE TAKEN YESTERDAY, DURING THE DAY AND DURING THE NIGHT. I WOULD LIKE TO KNOW WHERE YOU TOOK THE NOURISHMENT AND IF IT WAS COMBINED WITH OTHERS DURING THE LAST 3 DAYS. PLEASE, ALSO INCLUDE ALL NOURISHMENT TAKEN OUT OF THE HOME.

FOOD CATEGORIES	FOOD	CONSUMED (24H EARLIER)			CONSUMED (48H EARLIER)			CONSUMED (72H EARLIER)		
		Yes	Nbr of times	No	Yes	Nbr of times	No	Yes	Nbr of times	No
**BUILDER FOOD**										
**VEGETABLE-LIKE**	GROUNDNUTS									
	BEANS (black, red or white)									
	NIEBE									
	NUTS									
	SOY									
	PEA									
**MILK AND MILK PRODUCTS**	CHEESE									
	ARTIFICIAL MILK									
	MATERNAL MILK									
	YOGHOURT/KOSSAM									
**ANIMAL PROTEIN**	SEA ANIMALS (prawns, crabs, sea snails…)									
	EDIBLE INSECTS (grasshoppers, cricket, termites, caterpillar…)									
	FISH									
	CHICKEN, DUCK									
	MEAT - beef, goat, lamb, pork									
**ENERGISING FOOD**										
**CEREALS**	WHEAT									
	CORN									
	MIL									
	PATES ALIMENTAIRES									
	RICE									
	SORGHUM									
	MORINGA									
**ROOTS AND TUBERS**	YAM<br>PLANTAIN									
	COCOYAM									

	CASSAVA									
	SWEAT POTATO									
	IRISH POTATO									
	TARO									
**PROTECTIVE FOOD**										
**VEGETABLES**	CABBAGE									
	BITTER-LEAF (ndolè)									
	TUBER LEAVES (zoom, Folon, …)									
**FRUITS-LIKE VEGETABLES**	GARDEN EGG, UCUMBER, SQUASH, PUMPKIN									
	CARROTS, GREEN PEPPER									
	OCRO									
	ONION									
	HOT PEPPER									
**FRUITS**	PINEAPPLE, MANGO									
	PEAR, PLUM									
	BANANA									
	ORANGE, LEMON, GRAPEFRUIT									
	PAPAYA/POPO									
	WATER MELON									
	TOMATO									

NB : Please mention it, if there is any food type, whether solid, semi solid or liquid that you may have consumed which is not mentioned above.

Interviewer's observations:
……………………………………………………………………………………………
……………………………………………………………………………………………

Interviewer (Name):………………………………

Signature :

Interviewer (Name):………………………………

Signature :

QUESTIONNAIRE

STUDY TITLE : Corrélation entre la réponse immuno-virologique et le profil nutritionnel des patients infectés par le VIH sous traitement dans la région Est-Cameroun

Numéro : ……………………………. FOSA : ……………………………….…   Nom :………………………………..... Prénoms :…………………………………   Date de Naissance (jj/mm/aaaa) :…………………… Age (en année ou mois révolus) :……......   Sexe : ◻ M ◻ F ; si F **Enceinte** : ◻ Oui ◻ Non   Profession :…………………………….…. Tel :…………………………...   Région : ……………… Département :……………….... Ville de résidence:………………….   Quartier:…………………………... Nombre d'enfants :……………………………………   Nombre d'enfants VIH + :……………………Hôpital de visite :…………………………………
Poids (kg) actuel:……………… Poids habituel…………… Taille (cm) : ………………..

1. **Statut éducationnel** : ◻ Pas de scolarisation ◻ Primaire
   ◻ Secondaire 1er cycle ◻ Secondaire 2ème cycle ◻ Universitaire

2. **Statut matrimonial** : ◻ Célibataire ◻ Concubinage ◻ Marié
   ◻ Divorcé/séparé ◻ Veuf/veuve
3. **Groupe ethnique** : …………………………………….

4. **Source d'eau à boire** : ◻ Protégée : (robinet, forage, minérale)
   ◻ Non protégée (puits, marigots, eau de pluie)

5. **Consommation d'alcool** : ◻ Occasionnelle ◻ Régulière ◻ Jamais

6. **Fumeur** : ◻ Oui ◻ Non

Si oui nombre de cigarettes par jour : ◻ <5 ◻ 5-10 ◻ 10-20 ◻ >2

7. **Pensez-vous que votre Statut économique soit** : : ◻ < 500frs ◻ 500 - 1000
   ◻ 1000 - 5000 ◻ > 5000 frs
8. **Avez-vous déjà bénéficié d'un counseling nutritionnel** (dans le cadre de la prise en charge du VIH) ◻ oui ◻ non ◻ Autre situation :………………………………….
   Si oui, combien de séances de counseling nutritionnel avez-vous reçus :
   - < 3 mois ? ………………………………………..
   - 3 - 6 mois ? ....
   - > 6 mois ? ……………………………………………

9. **Nombre de repas/jour (en moyen), gouters inclus.**
   ◻ 1 ◻ 2 ◻ 3 ◻ 4 ◻5 ◻ 6 ◻>7

10. **Avez-vous déjà eu un épisode de malnutrition** ? ◻ Oui ◻ Non ◻ Ne sait pas
    Si oui quand ? …………………………………………………
    Quel type de malnutrition : ◻ insuffisance pondérale ◻ Surpoids ◻ Obèse

11. **Examen Clinique** :

☐ Fièvre ☐ Nausées ☐ Anorexie (perte d'appétit)
☐ Diarrhée (☐ Oui ☐ Non Si oui, nombre d'épisode par jour………)
☐ Vomissement (☐ Oui ☐ Non Si oui, nombre d'épisode par jour………)

12. **Œdèmes** : ☐ Absent ☐ Présent Si présent, localisation :

☐Pieds ☐ Pré tibial ☐ Mains ☐ Visage ☐ Généralisée

13. **Autre symptômes et signes cliniques** : ……………………………………………………………

14. **Autre pathologies** : ☐ Oui ☐ Non

Si oui: ☐ Tuberculose ☐ VHB ☐ VHC ☐ Paludisme ☐ Fièvre Typhoïde
☐ Diabète Autres……………………………………………………………

15. **Stade OMS de la maladie VIH** : ☐ 1 ☐ 2 ☐ 3 ☐ 4

16. **Avez-vous perdu du poids durant les 3 derniers mois** ? ☐ Oui ☐ Non ☐ Ne sait pas

17. **Avez-vous déjà pris** :

- Vit A : ☐ Oui ☐ Non
- Autres micronutriments/ ☐ Oui ☐ Non

18. **Traitement ARV** :

Date du 1er diagnostic VIH …… /…… /……
Mode d'infection ☐ Vertical ☐ Horizontal

Traitement VIH 1 : ☐ ……………………………………….. du …../…../….. au ..…/…../…..
Traitement VIH 2 : ☐ ……………………………………….. du …../…../….. au ..…/…../…..
Traitement VIH 3 : ☐ ……………………………………….. du …../…../….. au ..…/…../…..

MAINTENANT, JE VOUDRAIS VOUS POSER DES QUESTIONS SUR LES ALIMENTS QUE VOUS POURRIEZ AVOIR PRIS HIER, DURANT LE JOUR OU LA NUIT. JE VOUDRAIS SAVOIR VOUS AVEZ PRIS CET ALIMENT MEME S'IL EST COMBINE AVEC D'AUTRES ALIMENTS CES 3 DERNIERS JOURS

SVP, INCLURE AUSSI TOUS LES ALIMENTS CONSOMMES EN DEHORS DE LA MAISON.

CATÉGORIE D'ALIMENTS	ALIMENTS	COSOMMÉ (24 h)			COSOMMÉ (48 h)			COSOMMÉ (72 h)		
		OUI	Nbr de fois	NON	OUI	Nbr de fois	NON	OUI	Nbr de fois	NON
**ALIMENTS CONSTRUCTEURS**										
**LÉGUMINEUX**	ARACHIDES									
	HARICOT (noir, rouge ou blanc)									
	NIEBE									
	NOIX									
	soja									
	POIS									
**LAIT ET PRODUITS LAITIERS**	FROMAGE									
	LAIT ARTIFICIEL									
	LAIT MATERNEL									
	YAOURT/kossam									
**PROTÉINES ANIMALES**	FRUITS DE MER (crevettes, crabes, escargots de mer…)									
	INSECTES COMESTIBLES (criquets, termites, chenilles…)									
	POISSON									
	POULET, CANARD									
	VIANDE de bœuf, chèvre, agneau, porc									
**ALIMENTS ÉNERGÉTIQUES**										
**CÉRÉALES**	BLÉ									
	MAÏS									
	MIL									
	PATES ALIMENTAIRES									
	RIZ									
	SORGHO									
	MORINGA									
	IGNAMES<br>PLANTAINS									

RACINES ET TUBERCULES	MACABO									
	MANIOC									
	PATATE DOUCE									
	POMMES DE TERRE									
	TARO									
**ALIMENTS PROTECTEURS**										
LÉGUMES-FEUILLES	CHOU									
	Ndolè									
	FEUILLES DE TUBERCULE (zoom, Folon, …)									
LÉGUMES-FRUITS	AUBERGINE, CONCOMBRE, COURGES, CITROUILLE									
	CAROTTES, POIVRON									
	GOMBO									
	OIGNON									
	PIMENT									
FRUITS	ANANAS, MANGUE									
	AVOCAT, PRUNE									
	BANANE									
	ORANGE, CITRON, PAMPLEMOUSSE									
	PAPAYE									
	PASTÈQUE									
	TOMATE									

NB : Veuillez nous dire s’il y a n’importe quels autres aliments solides, semi-solides, ou mous non mentionné que vous avez consommé.

Interviewer’s observations:
……………………………………………………………………………………………
……………………………………………………………………………………………

Enquéteur (Nom):………………………………………

Signature :

Enquéteur (Nom):………………………………………

Signature :
